# Massive Upper Gastrointestinal Bleeding Caused by Diffuse Large B-Cell Lymphoma

**DOI:** 10.1155/2016/5079709

**Published:** 2016-10-24

**Authors:** O. Telci Caklili, H. H. Mutlu, Y. Colak, E. Ozturk, D. Kosemetin Dover, I. Tuncer

**Affiliations:** ^1^Kocaeli State Hospital, Internal Medicine, Kocaeli, Turkey; ^2^Family Medicine, Istanbul Medeniyet University Goztepe Training and Research Hospital, Istanbul, Turkey; ^3^Gastroenterology, Istanbul Medeniyet University Goztepe Training and Research Hospital, Istanbul, Turkey; ^4^Hematology, Koc University Medical School, Istanbul, Turkey; ^5^Pathology, Istanbul Medeniyet University, Istanbul, Turkey

## Abstract

Massive upper gastrointestinal bleeding is a life-threatening emergency which needs urgent intervention. Hematological malignancies are very rare causes of this type of bleeding and they usually originate from duodenum. In this case we present a gastric diffuse large B-cell lymphoma (DLBCL) causing massive upper gastrointestinal system bleeding. A 77-year-old male patient was admitted to emergency clinic with hematemesis and hematochezia. In physical examination patient was pale and sweaty; his vitals were unstable with a heart rate of 110 per minute and a blood pressure of 90/50 mmHg. His hemoglobin level was found 7.5 g/dL and he was transfused with one unit of packed red blood cells. After his vitals were normalized, gastroscopy was performed showing mosaic pattern in corpus and antrum mucosa and multiple ulcers in various sizes, largest being approximately 2 cm in diameter, higher than mucosa covered with exude mostly on corpus and large curvature. Biopsy results were reported as DLBCL. Gastric mucosa is involved in most of the DLBCL cases. Although not listed as a common cause of massive gastrointestinal bleeding DLBCL can cause life-threatening situations mostly because of its malignant nature.

## 1. Introduction

Massive upper gastrointestinal bleeding is a life-threatening emergency which needs urgent intervention. Most common etiological causes are gastric and/or duodenal ulcers esophagogastric varices with or without portal hypertensive gastropathy, Dieulafoy's lesions, and aortoenteric fistulas [1]. Hematological malignancies are very rare causes of this type of bleeding and they usually originate from duodenum [2]. In this case we present a gastric diffuse B-cell lymphoma causing massive upper gastrointestinal system bleeding.

## 2. Case

**Figure 1:**
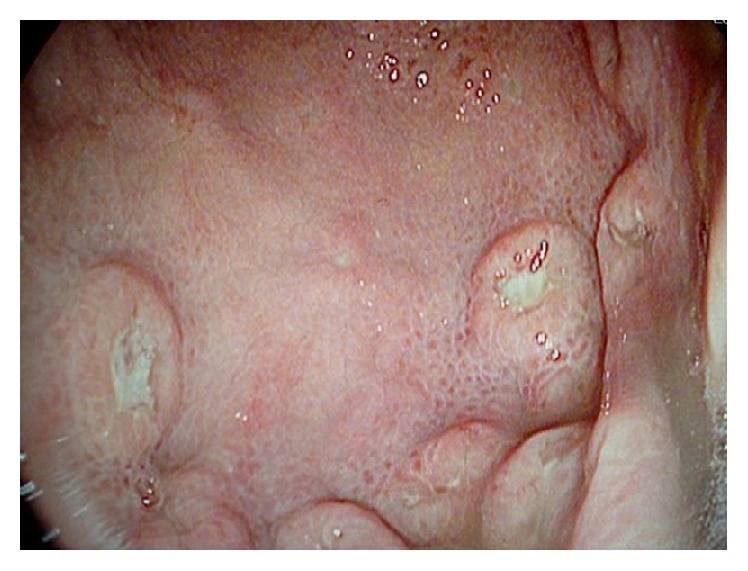
Endoscopic image of the stomach.

**Figure 2:**
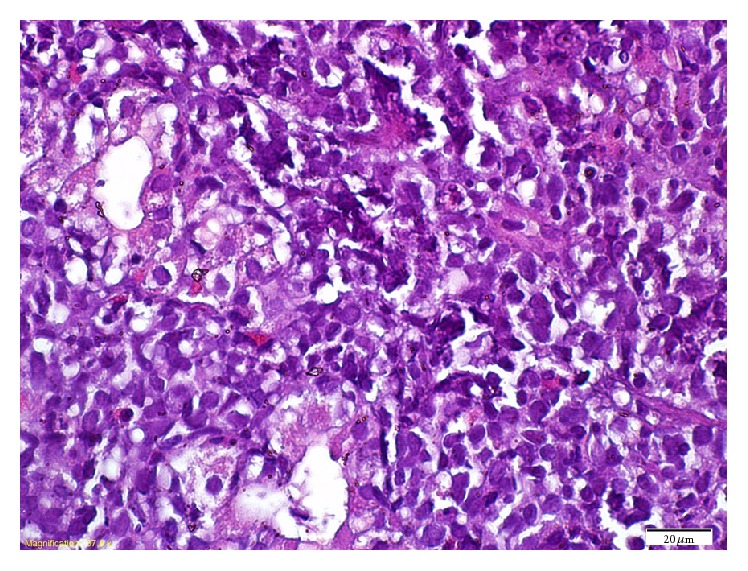
Pathology of the stomach.

A 77-year-old male patient was admitted to emergency clinic with hematemesis and hematochezia. There was no history of antiaggregant, anticoagulant, or nonsteroid anti-inflammatory drug use. In physical examination patient was pale and sweaty; his vitals were unstable with a heart rate of 110 per minute and a blood pressure of 90/50 mmHg. His hemoglobin level was found to be 7.5 g/dL and he was transfused with one unit of packed red blood cells. After his vitals were normalized, gastroscopy was performed showing mosaic pattern in corpus and antrum mucosa and multiple ulcers in various sizes, largest being approximately 2 cm in diameter, higher than mucosa covered with exude mostly on corpus and large curvature (Figure 1). Biopsy results were reported as diffuse large b-cell lymphoma (Figure 2). Patient was referred to a hematology clinic for rituximab plus 70% cyclophosphamide, doxorubicin, vincristine, and prednisone treatment (reduced R-CHOP). Patient was lost to follow-up.

## 3. Discussion

Diffuse large B-cell lymphoma is the most common type of lymphoma [3]. Although it can have various presentations, symptoms of nodal involvement are the most frequent. Gastric mucosa is involved in most of the cases with extra nodal involvement [4].

Treatment of such lymphoma has changed in the past years. Surgery was considered as first-line treatment until recently. Nowadays it is not preferred unless there is need for urgent surgery for severe perforation or bleeding or palliative treatment [5]. Irradiation is also chosen as add-up therapy in selected cases [6]. Our patient was referred for reduced R-CHOP therapy due to his age. Reports have shown increased survival with this protocol in elderly patients [7].

In English written literature we found one case similar to ours. Shum et al. have presented a case with diffuse large B-cell gastric lymphoma who has arrived to emergency clinic with shortness of breath [8]. In another case by Stratigos et al. a 42-year-old man with diffuse large B-cell of the duodenum was admitted to emergency clinic because of massive hematemesis [9].

Although not listed as a common cause of massive gastrointestinal bleeding DBCL can cause life-threatening situations mostly because of its malignant nature.
